# Multiple health behaviors before and after a cancer diagnosis among women: A repeated cross‐sectional analysis over 15 years

**DOI:** 10.1002/cam4.2924

**Published:** 2020-03-05

**Authors:** Daniel N. Tollosa, Elizabeth Holliday, Alexis Hure, Meredith Tavener, Erica L. James

**Affiliations:** ^1^ School of Medicine and Public Health University of Newcastle Newcastle NSW Australia; ^2^ Hunter Medical Research Institute Newcastle NSW Australia

**Keywords:** cancer survivors, multiple health behaviors (MHB), survivorship period

## Abstract

**Background:**

Cancer diagnosis may be a cue for health behavior change. Previous research that assessed the impact of a cancer diagnosis on multiple health behavior (MHB) change is limited by small sample size, cross‐sectional study design, and a focus on individual rather than multiple behaviors. This study investigated the impact of a cancer diagnosis on compliance with MHB recommendations.

**Methods:**

Data from Australian Longitudinal Study on Women's Health (ALSWH) were utilized. Compliance with MHB was assessed by cancer survivorship period; 0‐3 years pre‐diagnosis, 0‐3 years postdiagnosis, 4‐12 years postdiagnosis, and compared to controls. A MHB score based on the WCRF/AICR guidelines was calculated for six behaviors (physical activity, smoking, alcohol, BMI, fruit, and vegetable intake); scores ranged from 0 to 6, with a higher score indicating higher compliance. Generalized estimating equation (GEE) was used for statistical analysis.

**Results:**

Participants comprised 7585 women from the 2001 ALSWH survey, of whom 2285 developed cancer during 15 years of follow‐up. Compared to controls, the mean MHB score was slightly lower (Mean Difference (MD) = −0.015, *P* > .05) in survivors pre‐diagnosis, after adjusting for confounders; however, the compliance score increased during postdiagnosis, with the mean difference score being significantly higher in recent survivors (0‐3 years post diagnosis; MD = 0.055, *P* < .01). Likewise, within cancer survivors, the mean compliance score significantly increased 0‐3 years postdiagnosis (MD = 0.07, *P* < .05) compared to pre‐diagnosis, but this significant improvement was not maintained over the longer term (MD = 0.037, *P* > .05).

**Conclusion:**

In this sample, survivors had higher MHB score than controls. A cancer diagnosis may provide a teachable moment for health behavior change in the period immediately following diagnosis, but this effect was not sustained during longer‐term survivorship.

## Graphical abstract

Cancer survivors may adopt healthy behaviors after diagnosis, suggesting a cancer diagnosis could be a cue for adopting health behavior. However, in this study, a cancer diagnosis appeared to provide a teachable moment for health behavior change in the period immediately following diagnosis but the effect was not sustained during longer‐term survivorship.

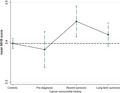

## 1 INTRODUCTION

More people are living with cancer than ever before, explained by early detection, improved treatment, and an aging population.^1^ Globally there are 43.8 million cancer survivors (those with a history of cancer) who are within 5 years of a cancer diagnosis.^2^

Despite improvements in survival, a cancer diagnosis is associated with increased risk of developing other chronic diseases, and deteriorating quality of life.^3^, ^4^ Health behaviors (eg, physical activity, healthy eating, not smoking, limiting alcohol consumption, and weight management) are salient practices related to a reduced risk of initial cancer, cancer recurrence and the likelihood of developing other chronic diseases including diabetes and cardiovascular disease.^5^

Cancer survivors report being highly motivated, receptive to messages about lifestyle choices^6^, ^7^ and willing to adopt healthy behaviors following their cancer diagnosis.^8^ This has led to the hypothesis that cancer diagnosis represents a “teachable moment” for health behavior change,^8^, ^9^, ^10^ regardless of age at diagnosis (eg, for physical activity^11^). However, empirical studies of this hypothesis are inconsistent, with some providing support,^6^, ^8^, ^12^, ^13^ whilst others conclude that cancer survivors demonstrate behaviors similar to the general population.^14^, ^15^, ^16^, ^17^ The teachable moment is also not supported by studies finding that many cancer survivors continue risky behaviors such as smoking^18^ and insufficient physical activity^19^ after diagnosis. However, these results derive largely from cross‐sectional studies that cannot establish a temporal relationship between cancer diagnosis and health behavior change. Longitudinal studies allow assessment of temporal relationships, but are scarce and largely limited to assessment at two time points: before and after a cancer diagnosis.^20^ Other limitations include grouping all survivors, regardless of time since diagnosis^21^, ^22^ and only including recently diagnosed survivors.^23^

MHB is associated with improved overall survival and quality of life in cancer survivors.^1^ The combined effect of MHB is also potentially greater than that of each individual behavior.^24^ However, to the best of our knowledge, longitudinal change in MHB has not been investigated in cancer survivors. Therefore, this study aimed to assess the temporal effect of a cancer diagnosis on adherence to MHB, consistent with six of the World Cancer Research Fund/ American Institute for Cancer Research (WCRF/AICR) lifestyle recommendations.^1^ This study addressed the following research questions:

- Do cancer survivors have lower MHB scores than controls, prior to their cancer diagnosis?
- Does the MHB score for cancer survivors as a group, improve after they are diagnosed in comparison to the pre‐diagnosis period?
- Is there a difference in MHB scores between recent (≤3 years) and long‐term (>3 years) survivors?
- Is there a difference in MHB scores between all survivors and controls?

## 2 METHOD

### 2.1 Study design

This was a repeated cross‐sectional analysis using prospective data from the Australian Longitudinal Study on Women's Health (ALSWH). The ALSWH began in 1996 (survey 1), with follow‐up surveys conducted approximately every 3 years.

### 2.2 Participants

The current study used ALSWH data from women born 1946‐1951, aged 45‐50 years at survey 1, 1996 (N = 13 714). Women participating in ALSWH are broadly representative of women in the Australian population; however, women in ALSWH were more educated compared to women in the general population,^25^ thus the reported health behaviors may be slightly overestimated. The ALSWH sampling frame was the Medicare database (then known as the Health Insurance Commission), which contains the name and address details of all Australian citizens and permanent residents.

**Figure 1:**
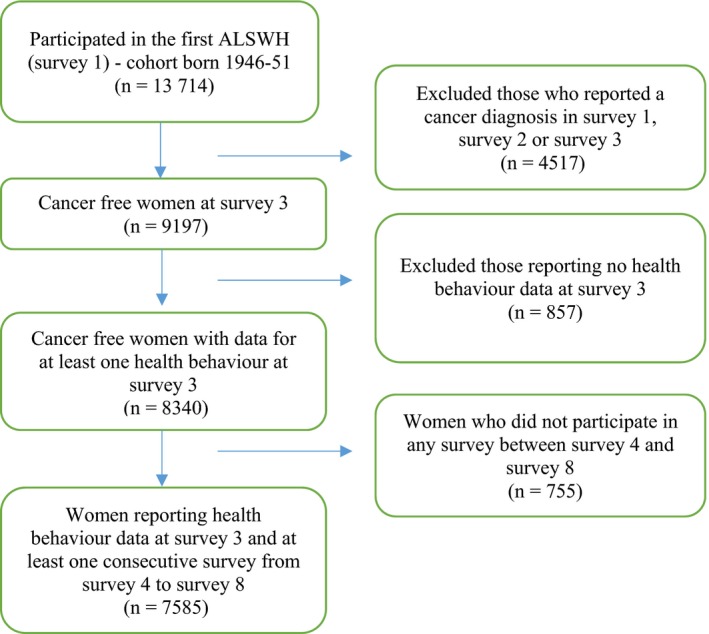
Selection of participants from Australian Longitudinal Study on Women Health (ALSWH), cohort born 1946‐1951, for the current study

Complete data for lifestyle behaviors were available from survey 3, 2001, when women were aged 50‐55 years (N = 8340). Therefore, survey 3 was the “baseline” for this study, which included only women who had reported no cancer history by that date. Women who answered “yes” to the following questions at survey 4 (2002‐2004), survey 5 (2005‐2007), survey 6 (2008‐2010), survey 7 (2011‐2013) or survey 8 (2014‐2016) were classified as *cancer survivors* and treated as an open cohort group: “In the past 3 years, have you been diagnosed or treated for: breast, cervical, lung, bowel (colorectal), skin (including melanoma), or other cancer” (Figure 1). *Controls* were defined as those who did not report a cancer diagnosis in any subsequent ALSWH survey from survey 1 in 1996. To be included, participants needed to respond to at least one ALSWH survey between surveys 4 and 8, and have baseline data (from survey 3, 2001) for at least one health behavior.

### 2.3 Measures

#### 2.3.1 Lifestyle behaviors

Outcome variables for this study comprised six health behaviors, reflecting women's health behavior at the time of survey: Body Mass Index (BMI), physical activity, alcohol use, cigarette smoking, fruit intake, and vegetable intake. For each outcome variable, a binary variable was created to denote adherence with the WCRF/AICR recommendations.^1^

Body Mass Index (BMI) was calculated using responses to survey questions: “How much do you weigh?” and “How tall are you without shoes?” BMI scores of 18.5‐24.99 kg/m^2^ were classified as meeting the healthy weight recommendation.

Validated Active Australia survey^26^ items for adults were incorporated into ALSWH surveys to measure self‐reported physical activity. At the survey, women were asked to report the total hours of activity (in 10 minutes bouts) in the past week: light intensity (eg, walking briskly), moderate intensity (eg, tennis, moderate exercise classes) and vigorous intensity (eg, competitive sport, swimming). The Metabolic Equivalent Task (MET) score, adapted from the compendium of physical activities (ie, 3.3 METs for light intensity, four METs for moderate‐intensity and 7.5 METs for vigorous leisure activities)^27^ was used to compute the total Physical Activity (TPA) score for women at each survey. Adherence to physical activity was defined a TPA score of ≥600 MET minutes/week.

For alcohol consumption, women were asked “How often do you usually drink alcohol?” Women who reported any alcohol intake were asked to quantify the amount in standard drinks (SD), ranging from “1 or 2 drinks per day” to “9 or more drinks per day”. Since the ALSWH data did not allow categorization to the recommended ≤1 SD per day for cancer survivors we used the closest category, that is, ≤2 SD to define adherence.

Smoking status was measured using the question “How often do you currently smoke cigarettes or any tobacco products?” Women who did not currently smoke were defined as meeting the WCRF/AICR smoking recommendation regardless of their history of smoking.

Fruit and vegetable consumption were assessed using a validated semi‐quantitative food frequency questionnaire (FFQ) known as the Dietary Questionnaire for Epidemiological Studies version 2,^28^ administered in the 2001 and 2013 survey. Total daily servings of fruit and vegetables were calculated by summing the mass of all fruits, vegetables, and fruit or vegetable juices reported in the FFQ. Total mass was divided by 150 g for fruit, 125 g for fruit or vegetable juices (added to fruit serves), and 75 g for whole vegetables (except 30 g serves for avocado, added to vegetables), consistent with the Australian Guide to Healthy Eating.^29^ In other ALSWH surveys (2004, 2007, 2010, and 2016), fruit and vegetable intake was assessed by asking: “How many pieces of fresh fruit do you usually eat per day? (1 piece of fruit refers to 1/2 cup of diced fruit, berries or grapes)” and “How many serves of vegetables do you usually eat per day? (a serve refers to 1/2 cup of cooked vegetables or a cup of salad vegetables)”. Adherence to fruit and vegetable intake was defined as fruit intake of ≥2 serves per day and vegetable intake of ≥5 serves per day.

MHB score, a count variable, was created from adherence scores to the six individual lifestyle behaviors using a summative index, ranging from zero (adherence to none of the recommendations) to six (adherence to all health behaviors). Women were excluded from the MHB scores if they did not have complete data for all individual health behaviors score, but were still eligible to participate in the study for the analysis of individual health behaviors.

#### 2.3.2 Cancer survivorship period

Given the possibility of multiple cancer occurrences across surveys, the first survey in which a woman reported a cancer diagnosis was defined as year of the first diagnosis. Survivorship time related to a cancer diagnosis was calculated by subtracting the age at year of first diagnosis from age at each survey (2004 to 2016), ranging from 12 years pre‐diagnosis to 12 years postdiagnosis. Survivorship time was used to classify the survivorship period as: *pre‐diagnosis* (≤3 years before diagnosis), *recent survivors* (≤3 years after reporting a cancer diagnosis) or *long‐term survivors* (4 −12 years after a cancer diagnosis). The main criteria for the survivorship period classification were the availability of data since ALSWH data are collected every 3 years. As an example, we illustrate the definitions of cancer survivorship period for women reporting a cancer diagnosis at ALSWH survey 6 (2010; Figure S1).

### 2.4 Covariates

The following covariates were included in this study: age, marital status (married/de facto, not married [combines separated/divorced/widowed/single]), occupation (paid job, no paid job), area of residence (urban, rural/remote), history of chronic illness comorbidities in the past 3 years (no condition, one to two conditions, or three or more of the nine possible conditions, collected at baseline and at each subsequent survey: diabetes, osteoarthritis, osteoporosis, heart disease, hypertension, stroke, asthma, depression, and anxiety), and education status (no formal education, certificate, or university degree). Women reported education status at two survey time‐points: survey 1 (1996) and survey 6 (2010), thus education status reported at survey 1 was used to define education for surveys 1‐5, while the response at survey 6 was used for surveys 6‐8. Detailed information about ALSWH survey methods and variables can be found at http://www.alswh.org.au/for-researchers/data.

A Directed Acyclic Graph (DAG) was constructed using DAGitty to depict assumptions regarding causal relationships between exposure, outcome, and covariates across consecutive surveys and identify potential confounders for inclusion in statistical models (Figure S2). Based on the DAG, an unbiased estimate of the relationship between cancer survivorship status and health behaviors at a given survey could be obtained by adjusting for prior (lagged) values of age, education status, comorbidities and MHB score, measured at the survey immediately prior.

### 2.5 Missing data management

Missing items for outcome variables (BMI, physical activity, alcohol consumption, smoking, fruit intake, and vegetable intake) were replaced using the response from either the preceding (first option) or subsequent survey (ie, forward or backward forecasting). We applied this technique to impute the missing data from the available longitudinal data, as recommended.^30^ Also, we did not find a significant difference (with versus without imputation) on the mean difference score of MHB but the confidence interval (precision) was relatively wide without imputation. The maximum missing proportion was 0.6%, for alcohol intake. For the MHB score, 1.3% of values were missing. For covariates, there were no missing values for age or number of comorbidities, but for the lagged score of MHB score and education status we used the second lagged score (ie, from the second preceding survey) if the first lagged score was missing. Complete case analysis was used since the proportion of missingness for all variables was less than 1.5%.

### 2.6 Statistical analysis

Data were analyzed using Stata (version 15) statistical software. Descriptive statistics (mean with standard deviation or frequencies with percent) was used to examine the characteristics of future cancer survivors and controls (cancer free women) at baseline (survey 3, 2001) for the current study. Groups were compared using *t* tests for continuous variables and chi‐squared tests for categorical variables. Percentages were adjusted for the area of residence because women in rural and remote areas were intentionally oversampled in ALSWH.

To describe adherence to health behavior recommendations, the proportion of participant's adherent to individual health behaviors, and the mean MHB score was calculated at each survey.

Generalized linear models (GLM) fitted in the Generalized Estimating Equation (GEE) framework were used to examine the impact of a cancer diagnosis (ie, exposure) on the MHB score, allowing for repeated measures on participants. Bivariate (unadjusted) and multivariate models (adjusted for covariate values from the previous survey, comprising age, education status, comorbidities, and MHB score) were fitted. Following estimation of the main model (ie, using controls as a reference group), the mean MHB scores were compared among cancer survivors using linear contrasts estimated using Stata's *lincom* function.

For individual behaviors, GLMs used a binomial response distribution and logit link, with results reported as odds ratios (OR) with 95% confidence intervals (CI). For the MHB score, a Gaussian distribution with identity link was used, with results reported as beta (β) coefficients with 95% CI. The quasi‐likelihood information criterion (QIC) was used to select the working correlation structure most appropriate for the data.

### 2.7 Sensitivity analysis

For the ALSWH cohort born from 1946 to 1951 (n = 13 714), 717 deaths were recorded between 1996 and 2014, of which cancer was at least one cause of death for 454 (63% of all 717 deaths). Data regarding tumor stage, indicating the severity of cancer, were limited; thus, we were unable to identify women with aggressive cancer that may influence compliance with lifestyle recommendations. However, we performed a sensitivity analysis to examine the effect of survival bias, that is, whether the sample of women who were still alive and therefore able to answer further ALSWH surveys were more compliant with health behaviors compared to those women who presumably had a more aggressive cancer and/or who were diagnosed at a later stage. Given the limitations imposed by lack of clinical data in the ALSWH dataset, we compared the results between the primary analysis (that included all survivors) and the group that excludes those women who died within 1 year since diagnosis and for whom cancer was a cause of death (n = 192 women).

The Bland‐Altman analysis was also used to quantify the mean difference between FFQ and short‐form questions related to fruit and vegetable intake from the 2001 ALSWH data.

## 3 RESULTS

### 3.1 Baseline participant characteristics

The current study included 7585 women who were cancer‐free at “baseline” (ALSWH survey 3), of whom 2285 reported a cancer diagnosis during the subsequent 15‐year period (ie, from survey 4 to survey 8). On average, the proportion of women diagnosed with cancer increased by 5.8% every 3 years (ie, each survey) between 2004 and 2016 (Table 1). At baseline (survey 3), cancer survivors were marginally older, fewer had obtained a university degree and lived in urban areas compared to controls (women who did not report a cancer diagnosis at any point during the study). There was no significant difference in marital status or the number of reported chronic diseases between cancer survivors and controls (Table 2).

**Table 1:** An open cohort of Australian female cancer survivors and controls aged 48‐55 y in 2001 and during the subsequent study period (2004‐2016)

Survey 3 (2001) (n = 7585)		Survey 4 (2004)	Survey 5 (2007)	Survey 6 (2010)	Survey 7 (2013)	Survey 8 (2016)
Baseline behavioral and anthropometric data from women who were cancer free	Survivors	501	501 + 543 = 1044	1044 + 468 = 1512	1512 + 380 = 1892	1892 + 393 = 2285
	Controls	5300	5300	5300	5300	5300

Note

The status at each survey reflects the presence or absence of a cancer diagnosis sometime during the 3 y preceding the survey year.

**Table 2:** Characteristics of future cancer survivors (reporting of a cancer diagnosis between 2004 and 2016 ALSWH surveys) and controls at baseline, in 2001

Characteristics		Survivors^a^ (N = 2285)		Controls (N = 5300)	*P*‐value
		Mean (SD)		Mean (SD)	
Age	N	52.1 (1.43)	N	52.0 (1.46)	<.001
		n (%)		n (%)	
Age group	2285		5300		
48‐51 y		1309 (57.1)		3205 (59.9)	.073
52‐55 y		976 (42.9)		2095 (40.1)	
Marital status	2175		4975		
Married/de facto		1817 (83.5)		4126 (82.9)	.315
Not married^b^		358 (16.5)		849 (17.1)	
Occupation	2018		4575		
No paid job		489 (23.0)		1077 (22.9)	.977
Paid job		1529 (77.0)		3498 (77.1)	
Area of resident	2166		4976		
Urban		1321 (61.0)		3234 (65.0)	<.001
Rural/remote		844 (39.0)		1741 (35.0)	
Education status^c^	2267		5259		
No formal education		342 (13.4)		938 (16.9)	.018
Certificate (intermediate/high school)		1150 (48.9)		2546 (46.1)	
Certificate (diploma/apprenticeship)		471 (20.8)		990 (19.6)	
University degree		304 (16.8)		782 (17.4)	
Chronic illness comorbidities	2176		5006		
None		1181 (55.4)		2747 (55.6)	.377
1‐2		880 (39.6)		2040 (40.3)	
≥3		115 (4.9)		219 (4.1)	

a Survivors refers to women who were cancer free at survey 3 but reported a cancer diagnosis in a subsequent survey from survey 4 to survey 8.

b Consists separated/divorced/widowed/single.

c Educational status measured at survey 1 (1996) and survey 6 (2010).

Table 3 shows results from the bivariate and multivariate linear regression models estimating the relationship between cancer diagnosis and MHB score.

**Table 3:** Relationship between cancer diagnosis and MHB score (with range 0‐6) among female cancer survivors and controls aged 48‐70 y, 2004‐2016 ALSWH surveys

Models		Cancer survivorship history				
		Controls	Survivors prior to diagnosis (≤3 y)	Recent survivors (0‐3 y post diagnosis)	Long‐term survivors (4‐12 y post diagnosis)	All survivors^a^
			β (95% CI)	β (95% CI)	β (95% CI)	β (95% CI)
Bivariate model^b^	a	Ref.	0.005 (−0.052, 0.061)	0.055 (0.005, 0.104)**	0.091 (0.052, 0.128)*	0.078 (0.046, 0.109)*
	b	—	Ref.	0.05 (−0.022, 0.122)	0.085 (0.021, 0.151)**	0.073 (0.011, 0.135)*
	c	—		Ref.	0.035 (−0.022, 0.094)	—
Multivariate model^c^	a	Ref.	−0.015 (−0.06, 0.31)	0.055 (0.014, 0.095)*	0.021 (−0.01, 0.053)	0.034 (0.01, 0.061)**
	b	—	Ref.	0.070 (0.011, 0.129)**	0.037 (−0.017, 0.091)	0.049 (−0.002, 0.053)
	c	—		Ref.	−0.033 (−0.082, 0.015)	—

a Includes recent and long‐term survivors.

b Unadjusted (only MHB score (dependent variable) and cancer survivorship history (covariate) included in the model).

c Adjustment was made for the following covariates: previous (lagged) score for adherence to MHB, age (as continuous), education status (categorical), number of reported comorbidities (categorical), area of resident (categorical) and the survey year. Results are shown as mean difference (β) with 95% CI.

* *P* < .01,

** *P* < .05.

**Figure 2:**
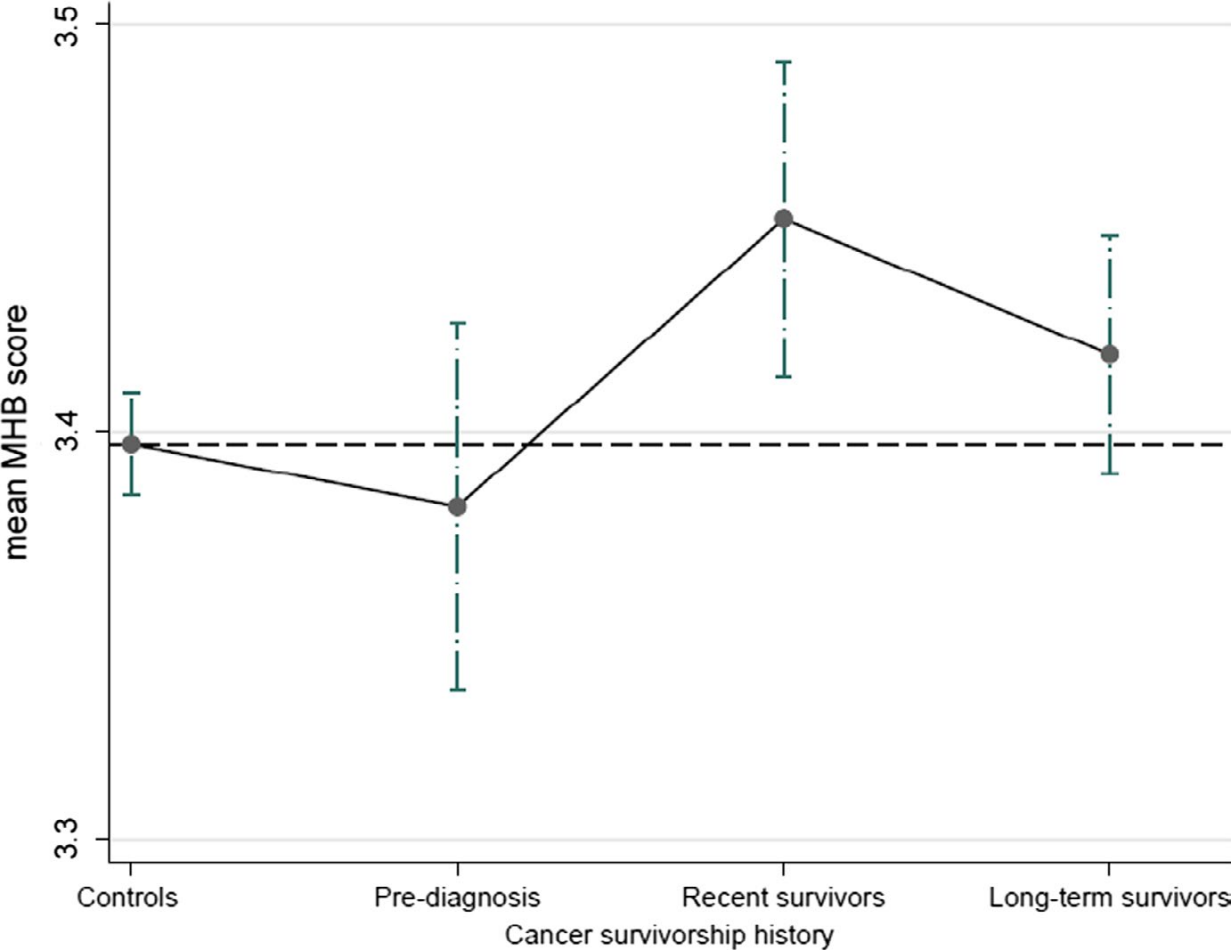
Multiple health behaviors (MHB) score (range 0‐6) by cancer survivorship history, data from open cohort of female cancer survivors and controls aged 48‐70 y, 2004 to 2016 Australian Longitudinal Study on Women Health (ALSWH) surveys. *Controls*—those never diagnosed with cancer; *Pre‐diagnosis*—those not yet but diagnosed to cancer within 3 y; *Recent survivors*—those survived cancer up to 3 y; *Long‐term survivors*—those survived cancer 4‐12 y. Results are shown as adjusted mean with 95% CI

In the adjusted model, during the three years preceding the cancer diagnosis, cancer survivors had a lower mean MHB score than controls (MD = −0.015, 95% CI: −0.06, 0.31) although the difference was statistically insignificant (*P* > .05). Following diagnosis, cancer survivors had significantly higher mean MHB score than controls (MD = 0.034, 95% CI: 0.01, 0.061), with the mean difference was significantly higher in recent survivors (MD = 0.055, 95% CI: 0.014, 0.06195) and slightly higher in long‐term survivors (MD = 0.021, 95% CI: −0.01, 0.05). An MHB score of 3.4 could be interpreted as the participant (women) achieving at least three out of the six recommended health behaviors (Table 3; Figure 2).

Within the survivorship group, the mean MHB score increased during postdiagnosis period from the period prior (up to 3 years) to the cancer diagnosis; however, the score was significantly higher in recent survivors (MD = 0.07, 95% CI: 0.011, 0.13) and thereafter dropped back to slightly higher but insignificant (MD = 0.037, 95% CI: −0.017, 0.091) when the survivorship period further increased. Moreover, compared to the recent survivors, the compliance score was slightly lower in long‐term survivors but the difference was not significant (*P* > .05).

Results were broadly similar in a sensitivity analysis excluding women who died within 1 year since diagnosis and for whom cancer was a cause of death. However, compared to the primary analysis (that includes all survivors), the mean difference was relatively higher in the cancer survivors—both during pre‐diagnosis and the recent survivorship period compared to the control group (Table S1).

#### 3.1.1 Adherence to individual health behaviors

In the adjusted model, the odds of adherence to health behaviors were not significantly improved during the survivorship period, from the period prior to diagnosis. However, a significant difference was observed in long‐term survivors with the odds of compliance being 15% higher (AOR = 1.15, 95% CI: 1.07, 1.24) and 11% higher (AOR = 1.11, 95% CI; 1.02, 1.19) than controls for physical activity and BMI, respectively. Cancer survivors were more likely to be nonsmokers (AOR = 1.32, 95% CI: 1.04, 1.47) than controls during the recent survivorship period (Table S2).

## 4 DISCUSSION

In this nationally representative sample of adult women (aged 45 to 70), with data collected over 15 years, we found no evidence that cancer survivors had significantly lower compliance with MHB than the cancer free group in the pre‐diagnosis period. Following a cancer diagnosis, MHB scores significantly improved compared to controls. Within the survivor group, the mean MHB score increased between pre‐diagnosis and recent survivorship and then decreased as women progressed further away from diagnosis.

Our hypothesis that the lifestyle behaviors of those who went on to be diagnosed with cancer would be less compliant than those who did not get cancer, was supported in the current study, although the difference was not statistically significant. Survivors perceiving early warning signs of cancer and adopting risk‐reducing health behaviors in the period near to diagnosis might explain the nonsignificant difference. During the immediate postdiagnosis period, cancer survivors reported healthier behaviors compared to the controls and compared to the pre‐cancer diagnosis period. This suggests a cancer diagnosis may prompt health behavior change following diagnosis, which could be associated with psychological attributes, such as a sense of control over the course of illness.^31^ Evidence shows healthy behaviors may help survivors to control cancer advancement, and prevent cancer recurrence or secondary cancer.^1^, ^32^ This finding is similar to other prospective studies,^12^, ^22^, ^33^ measuring individual behaviors (rather than a combined score) among recent survivors, that concluded cancer survivors make positive health‐related behavior changes after a cancer diagnosis.

The term “teachable moment” is often used to describe a particular event or circumstances thought to trigger individuals to adopt healthy behaviors that are risk‐reducing.^34^ In the current study, there was a significant improvement in MHB score immediately postdiagnosis from prior to diagnosis; but this initial improvement was not sustained. Rather, survivor's compliance decreased as time in survivorship increased. Similar to our finding longitudinal analyses by Broderick et al (2014)^9^ and Satia et al (2004)^22^ shows a cancer diagnosis is a key period for behavior change during early survivorship period. Conversely, other studies (eg, a longitudinal study by William et al 2013)^23^ found survivors' health behaviors (smoking, alcohol and physical activity) did not significantly improve from pre‐diagnosis to <4 years postdiagnosis, although there was some evidence of a transient improvement. Bidstrup et al (2013)^35^ also concluded a cancer diagnosis was not associated with improvements in lifestyle behaviors from pre to postdiagnosis. This discrepancy might be related to the sample size and the classification of a survivorship period in the studies. Overall, given the low guideline adherence rate on MHB in cancer survivors^36^, ^37^ and the role of health behaviors in reducing medical and psychological effects during the survivorship period,^38^ results from the current study suggest that appropriately timed advice and continuous support for survivors' behavior change during the course of the survivorship period are required. Moreover, lifestyle modification needs to be a core component of cancer survivorship care.

The recent National Comprehensive Cancer Network (NCCN) guideline also suggests the need for a considerable scope of behavior change interventions in the oncology context.^38^ Health behaviors are processes and practices embedded in social life and the possible compensatory health belief effect (ie, a belief that a risk of an unhealthy behavior compensated by a healthy behavior)^39^ makes changing many health behaviors together more difficult than changing any individually. Thus, MHB change interventions may require the involvement of different stakeholders (oncologists, primary care providers, psychologists, and other concerned bodies) whereby their collective actions could trigger a change sufficient to ensure guideline adherence among survivors.

Although this study did not explore clinician‐patient interactions, oncology clinicians are likely to be key players in increasing lifestyle recommendations to patients,^7^ however, their extent of involvement to discuss the importance of health behaviors with their patients was very low.^10^ Therefore, clinician ‐patient interaction needs to be strengthened throughout the survivorship period.

In the present study, the lower compliance score was observed among those who survive cancer 4‐12 years compared to the recent survivors (0‐3 survival years). This might suggest lifestyle change lasts for a short period, thus survivors could gradually relapse to risky health behaviors or to the level below the recommendation. Therefore, continuing efforts such as booster sessions following behavior change programs may require to strengthening survivors' capacity or self‐efficacy to cope with the social and psychological pressures over the survivorship period.

Concerning individual behaviors, despite improvements in adherence to physical activity and fruit intake the proportion of survivors who maintained a healthy body weight decreased over time (40% to 35%). Our findings are similar to Greenlee et al 2016^40^ who analyzed the US National Health Interview Survey (from 1997 to 2014), found that the prevalence of healthy weight decreased from 34% to 32% in cancer survivors. In Australia, nearly two‐thirds (63%) of adults, including women cancer survivors, were overweight or obese in 2014‐2015, steadily increased from 57% in 1995.^41^ These results could suggest the usual clinical and public health interventions for obesity control, which mainly focus on physical activity and diet, might not be sufficient for cancer survivors. Rather, comprehensive and multifaceted approaches that enable survivors to control their psychosocial and physical environment are needed.

The main addition of this study to the existing body of literature is the longitudinal nature of the data across multiple time points and the measurement of concurrent adherence to MHB. The effect size (ie, the observed difference between the groups) was small in the current study. The large sample size, and the fact that health behaviors are recommended for both survivors (eg, reducing recurrence and treatment effect) and cancer free person (eg, the prevention of chronic diseases including cancer itself)^1^ may contribute to the observed small difference between groups.

The following limitations need to be considered when interpreting and utilizing the results. First, due to the nature of ALSWH, participant's health behaviors and cancer history were self‐reported, potentially leading to under or over‐reporting. However, Stavrou et al (2011) reported more than 90% sensitivity and specificity for values of self‐reported cancer diagnosis from ALSWH surveys, using the Central Cancer Registry (CCR) as a gold standard.^42^ Secondly, we did not distinguish between cancer types that might affect the level of adherence. However, the sensitivity analysis excluding participants where cancer was one of the causes of death did not alter the main findings. Third, there is the potential for some misclassification bias, particularly in the direction of greater adherence, in relation to the alcohol, fruit and vegetable behaviors, because of the measurement tools. A result from the Bland‐Altman statistics showed that FFQ under‐represent fruit and vegetable intake by a mean of 2.2 serves (95% CI: −2.25, −2.19) compared to the short form question. Measuring multiple behavior change over time is complex and using a summative index method relies on the assumption of each behavior is equivalent and mutually exclusive. A recent publication by Shams‐White et al (2019) suggested a standardized scoring system for the WCRF/AICR guidelines, which consider half points for those who partially follow the guidelines.^43^ There are no published guidelines to assess what constitutes a clinically meaningful difference in a MHB score within or between groups. Further research assessing the relationship between MHB scores and cancer recurrence, co‐morbidity, cancer mortality and all‐cause mortality is required. Fourth, the number of participants in this study declined across the surveys, from 7585 in 2001 to 5680 in 2016, with potential participation bias, limiting generalizability. However compared to the other cohorts in ALSWH, retention of participants has been highest in the 1946‐51 cohort.^44^ Further, because only female cancer survivors' data were available for this study, the results may not be generalizable to other population groups. Fifth, due to lack of data we are unable to consider potential confounders for adherence to a healthy lifestyle such as cancer treatment received, cancer stage and information regarding whether or not survivors received advice and support throughout their survivorship period. Further studies investigating a change in compliance of MHB within each person over time and determine the level of MHB score with the associated clinical and psychological impact in cancer survivors are needed.

## 5 CONCLUSION

Women who survived cancer had higher compliance with MHB than controls, indicating that a cancer diagnosis may offer a teachable moment for improving health behaviors immediately postdiagnosis. However, this initial improvement was not sustained as survivorship years increased. Interventions designed to enhance the maintenance of positive behavior change over the long‐term are required.

## CONFLICTS OF INTEREST

The authors declare no conflict of interest.

## AUTHOR CONTRIBUTIONS

DNT, EH, AH, MT, and EJ conceptualized the study; DNT, AH and EH conducted the analysis; DNT prepared the draft manuscript. All authors contributed to and approved the final manuscript.

## Data Availability

Information about data access of the Australian Longitudinal Study on Women's Health is available at https://www.alswh.org.au/
