# Supplementary figures and images for: Multiple health behaviors before and after a cancer diagnosis among women: A repeated cross‐sectional analysis over 15 years
Source: Cancer Med. 2020 Mar 5;9(9):3224–33. doi: 10.1002/cam4.2924 (PMC7196049; doi:10.1002/cam4.2924)

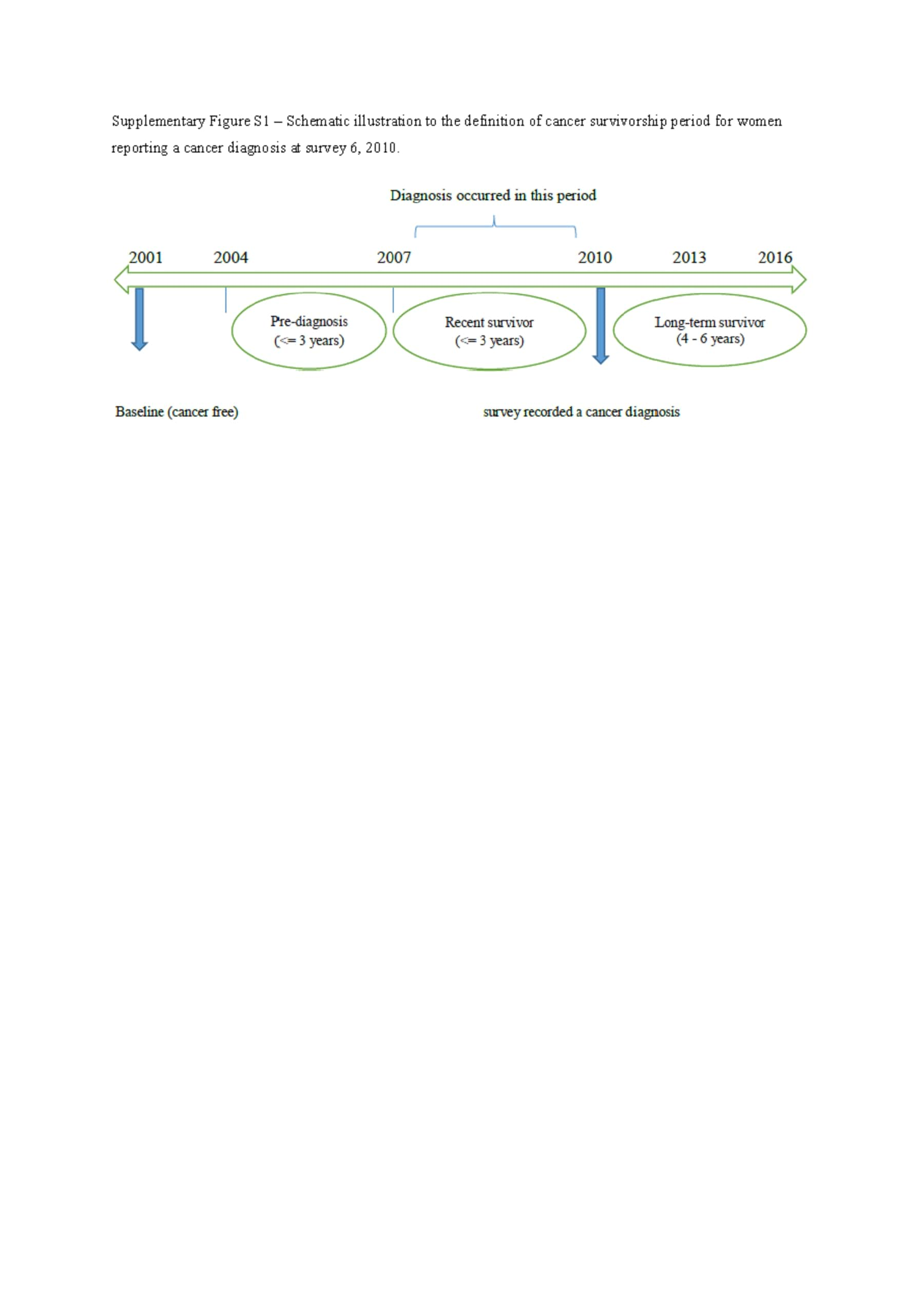

Supplement: Supplementary file 1 [file CAM4-9-3224-s001.tif]

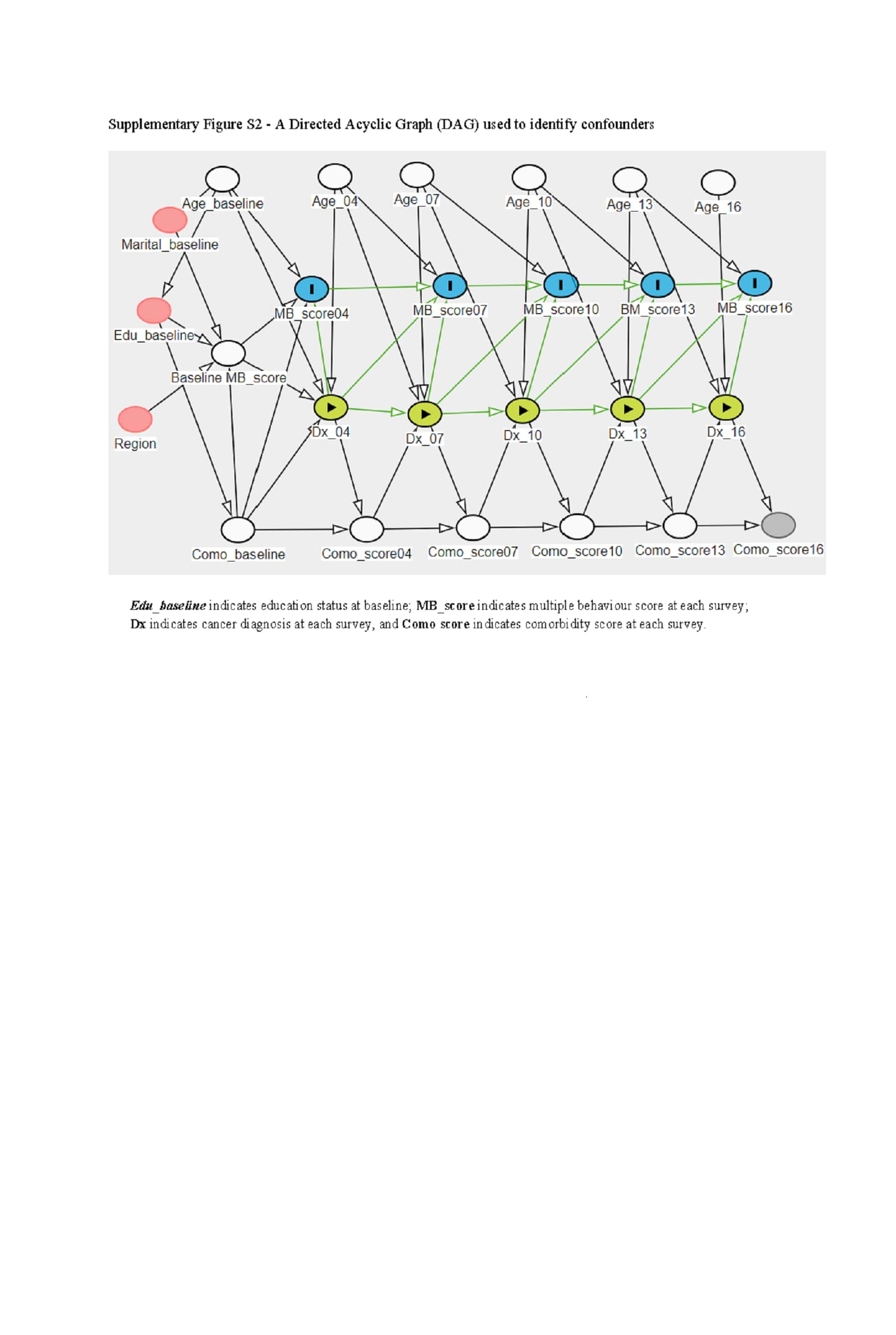

Supplement: Supplementary file 2 [file CAM4-9-3224-s002.tif]
