# Supplementary material for: Multiple health behaviors before and after a cancer diagnosis among women: A repeated cross‐sectional analysis over 15 years
Source: Cancer Med. 2020 Mar 5;9(9):3224–33. doi: 10.1002/cam4.2924 (PMC7196049; doi:10.1002/cam4.2924)
Supplement: Supplementary file 4 [file CAM4-9-3224-s004.docx]

Supplementary Figure S1 – Schematic illustration to the definition of cancer survivorship period for women reporting a cancer diagnosis at survey 6, 2010.


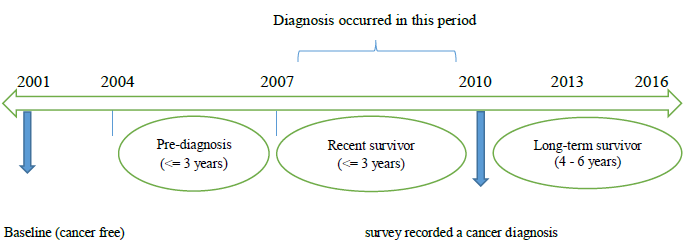


Supplementary Figure S2 - A Directed Acyclic Graph (DAG) used to identify confounders


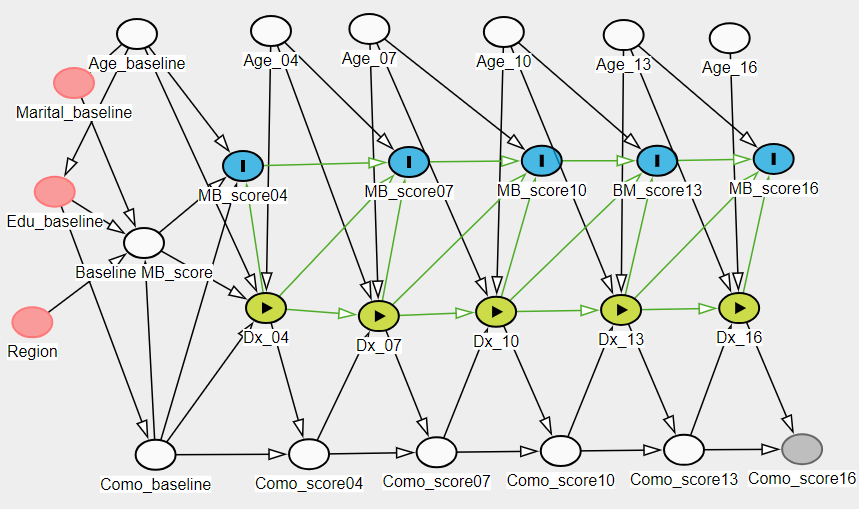


***Edu_baseline*** indicates education status at baseline; **MB_score** indicates multiple behaviour score at each survey;

**Dx** indicates cancer diagnosis at each survey, and **Como score** indicates comorbidity score at each survey.

Supplementary Table S1 - Results from sensitivity analysis, which excluded those women who died within 1 year since the reported year of cancer, and for whom cancer was one of the causes of death

| Models | | Cancer survivorship history | | | | | |
| --- | --- | --- | --- | --- | --- | --- | --- |
|  |  | Controls | Survivors prior to diagnosis (<=3 years) | Recent survivors (0-3 years post diagnosis) | Long-term survivors (4-12 years post diagnosis) | All survivors |  |
|  |  |  | β (95% CI) | β (95% CI) | β (95% CI) | β (95% CI) |  |
| Bivariate model**^α^** | a | Ref. | 0.014 (-0.043, 0.072) | 0.061 (0.011, 0.110)** | 0.091 (0.052, 0.128)* | 0.080 (0.048, 0.112)* |  |
|  | b | - | Ref. | 0.046 (-0.026, 0.119) | 0.076 (0.011, 0.142)** | 0.065 (0.003, 0.127) |  |
|  | c | - | | Ref. | 0.029 (-0.028, 0.088)* | - |  |
| Multivariate model^β^ | a | Ref. | -0.005 (-0.05, 0.041) | 0.057 (0.016, 0.098)* | 0.022 (-0.009, 0.053) | 0.034 (0.008, 0.061)* |  |
|  | b | - | Ref. | 0.063 (0.003, 0.0123)** | 0.027 (-0.026, 0.082) | 0.041(-0..011, 0.092) |  |
|  | c | - | | Ref. | -0.035 (-0.084, 0.013) | - |  |
| *P<0.01 **P<0.05 ^κ^includes recent and long-term survivors  ^α^Unadjusted (only MHB score (dependent variable) and cancer survivorship history (covariate) included in the model).  ^β^Adjustment was made for the following covariates: previous (lagged) score for adherence to MHB, age (as continuous), education status (categorical), number of reported comorbidities (categorical), area of resident (categorical) and the survey year.  (a) Compare cancer survivor’s compliance score, with their survivorship history (pre-diagnosis, recent and long-term survivorship) against the control group. (b) Compare recent and long-term survivors against their pre-diagnosis score. (c) Compare recent and long-term survivors. | | | | | | | |

Supplementary Table S2 - The effect of cancer survivorship on adherence to individual lifestyle behaviours, estimated using GEE with independent correlation structure and logit link function

| Adherence to the WCRF/AICR Health behaviours | Cancer survivorship history | | | | |
| --- | --- | --- | --- | --- | --- |
|  | Controls | Pre-diagnosis (0-3 years prior to diagnosis) | Recent survivors (0-3 years post diagnosis) | Long-term survivors (4-12 years post diagnosis) | All survivors^α^ |
|  |  | OR (95% CI) | OR (95% CI) | OR (95% CI) | OR (95% CI) |
| Physical activity (>=600 MET-minutes/week) | | | | |  |
| Model 1 | Ref. | 1.03 (0.93, 1.14) | 1.04 (0.95, 1.14) | 1.22 (1.14, 1.31)* | 1.15 (1.09, 1.22)* |
|  | - | Ref | 1.01 (0.89, 1.15) | 1.18 (1.05, 1.32)* | 1.12 (1.00, 1.24)** |
| Model 2 | Ref. | 1.04 (0.94, 1.16) | 1.08 (0.98, 1.18) | 1.15 (1.07, 1.24)* | 1.12 (1.06, 1.19)* |
|  | - | Ref. | 1.03 (0.90, 1.18) | 1.11 (0.96, 1.26) | 1.07 (0.95, 1.20) |
| Cigarette Smoking (not smoking at all) | | | | |  |
| Model 1 | Ref. | 1.09 (0.91, 1.30) | 1.21 (1.03, 1.42)** | 1.60 (1.39, 1.83)* | 1.43 (1.28, 1.59)* |
|  | - | Ref. | 1.11 (0.87, 1.39) | 1.46 (1.17, 1.81)* | 1.31 (1.07, 1.60)* |
| Model 2 | Ref. | 1.20 (0.99, 1.44) | 1.32 (1.04, 1.47)** | 1.15 (0.99, 1.33) | 1.19 (1.06, 1.33)* |
|  | - | Ref. | 1.04 (0.81, 1.33) | 1.01 (0.78, 1.29) | 1.02 (0.82, 1.28) |
| Alcohol use (<= 2 SD/day) | | | | |  |
| Model 1 | Ref. | 0.94 (0.81, 1.09) | 1.01 (0.89, 1.15) | 1.18 (1.06, 1.31)* | 1.11 (1.02, 1.21)** |
|  | - | Ref. | 1.08 (0.89, 1.29) | 1.25 (1.05, 1.48)* | 1.18 (1.01, 1.38)** |
| Model 2 | Ref. | 0.98 (0.85, 1.15) | 1.05 (0.91, 1.19) | 1.06 (0.95, 1.19) | 1.05 (0.97, 1.15) |
|  | - | Ref. | 1.08 (0.88, 1.32) | 1.16 (0.95, 1.41) | 1.12 (0.94, 1.33) |
| BMI (18.5-24.9 kg/m^2^) | | | | |  |
| Model 1 | Ref. | 1.02 (0.92, 1.13) | 1.01 (0.92, 1.11) | 1.00 (0.94, 1.08) | 1.00 (0.95, 1.06) |
|  | - | Ref. | 0.98 (0.86, 1.12) | 0.98 (0.87, 1.10) | 0.98 (0.87, 1.09) |
| Model 2 | Ref. | 1.03 (0.92, 1.14) | 1.06 (0.96, 1.16) | 1.11 (1.02, 1.19)* | 1.09 (1.02, 1.61)* |
|  | - | Ref. | 1.01(0.88, 1.17) | 1.04 (0.91, 1.20) | 1.03 (0.91, 1.17) |
| Fruit intake (>= 2 serves per day) | | | | |  |
| Model 1 | Ref. | 0.94 (0.85, 1.04) | 1.02 (0.93, 1.11) | 0.92 (0.86, 0.99) | 0.95 (0.90, 1.01) |
|  | - | Ref. | 1.03 (0.90, 1.17) | 1.09 (0.97, 1.22) | 1.06 (0.95, 1.19) |
| Model 2 | Ref. | 0.96(0.84, 1.09) | 1.05 (0.93, 1.17) | 1.00 (0.92, 1.08) | 1.02 (0.94, 1.09) |
|  | - | Ref. | 1.08 (0.91, 1.29) | 1.04 (0.89, 1.21) | 1.06 (0.92, 1.23) |
| Vegetable intake (>= 5 serves per day) | | | | |  |
| Model 1 | Ref. | 1.07 (0.90, 1.26) | 1.16 (1.01, 1.34)** | 1.11 (0.99, 1.24) | 1.12 (1.03, 1.23)* |
|  | - | Ref. | 1.12 (0.90, 1.40) | 1.22 (1.00, 1.51)** | 1.18 (0.97, 1.44) |
| Model 2 | Ref. | 1.12 (0.92, 1.36) | 1.12 (0.96, 1.30) | 1.01 (0.89, 1.15) | 1.05 (0.95, 1.18) |
|  | - | Ref. | 0.98 (0.76, 1.24) | 0.86 (0.66, 1.11) | 0.93 (0.74, 1.16) |
| Fruit (>= 2 serves per day) and vegetable intake (>= 5 serves per day) | | | | |  |
| Model 1 | Ref. | 1.10 (0.91 1.33) | 1.17 (1.01, 1.38)** | 1.13 (0.99, 1.28) | 1.14 (1.03, 1.27)* |
|  | - | Ref. | 1.07 (0.83, 1.36) | 1.18 (0.94, 1.49) | 1.14 (0.92, 1.41) |
| Model 2 | Ref. | 1.17 (0.95, 1.45) | 1.15 (0.96, 1.37) | 1.07 (0.92, 1.29) | 1.10 (0.97, 1.25) |
|  | - | Ref. | 0.95 (0.72, 1.25) | 0.85 (0.64, 1.13) | 0.91 (0.70, 1.17) |
| *P<0.01 **P<0.05 ^α^includes recent and long-term survivors OR – Odds Ratio  Model 1 – a bivariable model (only MHB score (dependent variable) and cancer survivorship history (covariate) included in the model. Model 2 – a multivariable (adjusted) model for the following covariates: previous (lagged) score for adherence to MHB, age (as continuous), education status (categorical), number of reported comorbidities (categorical), area of residence (categorical) and for time (i.e. the survey year). | | | | | |
